# The Immediate Response of Craniofacial Structures and Soft Tissue Periodontium to the 2-Hinged Expander Activated by Alt-RAMEC During the Growth Period: A Single-Center, Prospective, Comparative Study

**DOI:** 10.3390/jcm15082882

**Published:** 2026-04-10

**Authors:** Hatice Gökalp, Nuri Can Tanrısever

**Affiliations:** Department of Orthodontics, School of Dentistry, University of Ankara, Ankara 06560, Türkiye

**Keywords:** maxilla, malocclusion, angle class III, orthodontic appliances, palatal expansion technique, periodontal diseases

## Abstract

**Background/Objectives:** This study aimed to evaluate the immediate effects of a 2-hinged expander activated with the alternate rapid maxillary expansion–constriction (Alt-RAMEC) protocol on craniofacial structures and the soft tissue periodontium in adolescents with skeletal Class III malocclusion characterized by maxillary retrusion. **Methods:** Lateral cephalograms obtained at baseline (T0) and immediately after treatment (T1) from 15 adolescents (6 females, 9 males; mean ages 12.6–13.1 years) treated with a 2-hinged expander using a 9-week Alt-RAMEC protocol were analyzed. A control group consisted of 27 untreated Class III individuals (7 females, 20 males; mean ages 12.5–12.6 years). Sagittal and vertical skeletal, dental, and soft tissue measurements were assessed using a Cartesian coordinate system. Periodontal parameters of supporting teeth were evaluated at T0 and T1. Statistical analysis was performed using the Mann–Whitney U and Wilcoxon tests (*p* < 0.05). **Results:** Significant anterior maxillary displacement was observed in the treatment group compared with controls (*p* < 0.01), accompanied by increases in overjet and Wits appraisal (*p* < 0.05), while mandibular position remained unchanged. The upper lip advanced in accordance with skeletal changes (*p* < 0.05). Gingival index, bleeding index, and probing pocket depth increased significantly in supporting teeth (*p* < 0.05), whereas plaque index remained stable (*p* > 0.05). **Conclusions:** The 2-hinged expander combined with a 9-week Alt-RAMEC protocol induces immediate skeletal maxillary advancement in growing Class III patients with minimal dental compensation. Short-term periodontal changes suggest a transient inflammatory response associated with appliance therapy.

## 1. Introduction

Skeletal Class III malocclusion is a clinically significant dentofacial deformity characterized by a sagittal discrepancy between the maxilla and mandible, often associated with maxillary deficiency. Its prevalence varies across populations and is reported to be higher in certain ethnic groups [1]. If left untreated, skeletal Class III malocclusion may lead to functional impairments, esthetic concerns, and psychosocial difficulties, thereby emphasizing the importance of early and effective orthopedic intervention; during the growth period, such interventions aim to modify skeletal development, whereas in adulthood, when growth potential is limited, orthognathic surgery may be required to achieve optimal functional and esthetic outcomes [2,3].

The maxillary bone grows through regional remodeling and by stimulating growth sites within the cranial skeleton in response to functional demands in the craniofacial region. A discrepancy between maxillary and mandibular growth may result in skeletal disharmony and subsequent malocclusion. Skeletal Class III malocclusion associated with maxillary deficiency occurs when the maxilla is retrognathic relative to a normal or prognathic mandible [4,5].

Maxillary expansion is one of the oldest orthopedic approaches used to separate the nasomaxillary complex from the cranial base. It is commonly performed with removable or fixed expansion appliances in the management of Class III malocclusion characterized by maxillary retrusion, with a history extending over a century [6,7,8,9]. In 1860, Emerson C. Angell reported successful maxillary expansion prior to the advent of radiography, demonstrating that transverse separation could be achieved using a screw-based appliance applied to the supporting teeth [10]. Sutural maturation during early skeletal growth limits the ability to mobilize the maxilla orthopedically. Melsen demonstrated in human autopsy specimens that sagittal displacement of the midpalatal suture coincides with transverse palatal growth [11].

The palatine bone connects the cranial base through the sphenoid and the facial skeleton through the maxilla, and its articulations become increasingly interdigitated with age. Quantitative histological analyses have shown that the contact surface between the palatine bone and adjacent maxillary and sphenoidal structures enlarges over time. Sutural tissues respond to orthodontic forces in a manner similar to periodontal ligaments, and the magnitude of the reaction depends on the surface area involved. The extensive articulation surfaces in the palatomaxillary region make complete maxillary disarticulation by orthodontic forces difficult. However, orthodontic forces applied during adolescence can stimulate osteogenic responses in wide sutural areas. Haas reported that rapid maxillary expansion (RME) during adolescence facilitates forward and downward displacement of the maxilla due to circummaxillary sutural separation around the tuberosity region [12].

The primary objective of maxillary expansion is to reduce resistance within the palatomaxillary region to facilitate maxillary mobilization. Biederman and Chem emphasized that the center of rotation generated by RME influences the sagittal displacement of the maxilla [7]. Although RME reliably produces transverse skeletal effects by separating the midpalatal suture, its sagittal influence is less pronounced because tensile forces diminish toward circummaxillary sutures [6,7,8,13,14,15]. Prolonged expansion may therefore be required to achieve anterior maxillary movement, yet overexpansion may lead to adverse effects such as buccal non-occlusion, palatal mucosal irritation, and periodontal compromise of supporting teeth.

To enhance maxillary advancement, facemask (FM) therapy following maxillary expansion has been widely recommended for the treatment of skeletal Class III malocclusion with maxillary insufficiency [16,17,18,19,20,21,22,23,24]. However, treatment success is highly dependent on patient compliance, and limited cooperation may compromise skeletal outcomes [25,26].

In response to these limitations, the alternate rapid maxillary expansion and constriction (Alt-RAMEC) protocol was introduced to achieve more effective mobilization of the maxilla while minimizing excessive transverse expansion [25,27]. Clinical evidence suggests that Alt-RAMEC may produce 5–6 mm of maxillary advancement within approximately five months, exceeding the effects of conventional RME [27,28]. The biological rationale of Alt-RAMEC is based on the sequential application of tensile and compressive forces to the circummaxillary sutures through repetitive expansion–constriction cycles. These alternating forces are thought to disrupt the interdigitated sutural architecture, reduce skeletal resistance within the circummaxillary complex, and enhance sutural remodeling. As a result, the maxilla becomes more responsive to orthopedic forces, facilitating its anterior displacement. This effect is further enhanced by the use of a 2-hinged expander, which generates a posterior-lateral center of rotation and directs forces more effectively toward the circummaxillary sutures, thereby improving maxillary mobilization [25,27].

Unlike conventional maxillary expanders, which typically produce a center of rotation near the midpalatal suture and primarily generate transverse separation, the 2-hinged expander alters the center of rotation toward a more posterior region. This posterior displacement of the rotation center changes the direction of force transmission, allowing forces to be distributed more effectively to the circummaxillary sutures rather than being concentrated at the midpalatal suture. As a result, the 2-hinged expander may facilitate more efficient mobilization of the circummaxillary complex and enhance the anterior displacement of the maxilla.

Recent studies have evaluated Alt-RAMEC combined with tooth- and tissue-borne expanders, with or without facemask therapy, in the treatment of Class III malocclusion associated with maxillary retrusion [29,30,31,32,33,34].

Although the immediate alveolar effects of Alt-RAMEC have been investigated using cone-beam computed tomography [35,36], the immediate positional effect of the Alt-RAMEC protocol when used independently of facemask therapy—particularly in combination with a modified appliance design such as a 2-hinged expander—has not been clearly established in the current literature. Furthermore, while hard tissue periodontal responses to RME and Alt-RAMEC have been well documented [6,7,8,35,37,38,39], the short-term soft tissue periodontal response associated specifically with the combined use of Alt-RAMEC and a 2-hinged expander remains insufficiently investigated.

In addition, most previous studies have evaluated Alt-RAMEC in combination with facemask therapy, making it difficult to determine the independent effect of the expansion protocol itself. Moreover, the specific biomechanical and clinical effects of the 2-hinged expander have not been sufficiently investigated in comparison to conventional expanders. Therefore, current evidence is limited not only in isolating the skeletal effects of Alt-RAMEC without adjunctive protraction but also in clarifying the periodontal implications of this modified expansion approach.

Therefore, the aim of this prospective comparative clinical study was to evaluate the immediate effects of a 9-week Alt-RAMEC protocol using a 2-hinged expander on maxillary skeletal position and periodontal soft tissue response in adolescents with skeletal Class III malocclusion characterized by maxillary retrusion, and to compare these changes with growth-related variations observed in an untreated control group.

The working hypothesis of this study was that the Alt-RAMEC protocol combined with a 2-hinged expander would result in significant anterior maxillary displacement with minimal dentoalveolar effects and measurable short-term changes in soft tissue periodontal parameters.

## 2. Materials and Methods

### 2.1. Study Design and Ethical Approval

This prospective comparative clinical study was initially designed to include cone-beam computed tomography (CBCT) imaging. However, CBCT use in growing individuals was not approved by the Ethics Committee; therefore, lateral cephalometric radiographs were utilized. The study was conducted in accordance with the Declaration of Helsinki. Ethical approval was obtained from the Ethics Committee of the Faculty of Dentistry, Ankara University, Turkey (20 December 2019; No. 36290600).

Written informed consent was obtained from the parents or legal guardians of all participants.

An a priori power analysis was performed using G*Power software (version 3.1; Heinrich Heine University, Düsseldorf, Germany). The sample size was calculated based on the expected change in anterior maxillary position (A–VRL), which was considered the primary outcome variable of the study. Assuming a medium-to-large effect size (d = 0.80), a significance level of 0.05, and a statistical power of 80%, the minimum required sample size was calculated to be 20 participants. The study began with 20 patients; however, five were lost to follow-up due to pandemic-related restrictions, resulting in a final treatment sample of 15 patients.

### 2.2. Participants

The treatment group consisted of 15 consecutively treated adolescents (6 females, mean age 12.60 ± 1.42 years; 9 males, mean age 13.10 ± 1.92 years) with skeletal and dental Class III malocclusion characterized by maxillary retrusion and no transverse discrepancy.

Inclusion criteria were:

Skeletal Class III malocclusion due to maxillary retrusion (Nperp-A < −1 mm);

Normodivergent vertical pattern (FH/MP within normal limits);

Anterior crossbite with negative overjet;

CVM stages II–III;

Late permanent dentition.

Exclusion criteria included:

Absence of maxillary posterior teeth;

Congenital dental anomalies, impacted or ankylosed teeth;

Periodontal disease;

Previous orthodontic treatment;

Cleft lip and palate.

The control group comprised 27 untreated adolescents (7 females, mean age 12.50 ± 1.54 years; 20 males, mean age 12.60 ± 1.45 years) with skeletal and dental Class III malocclusion. Because withholding treatment may adversely affect prognosis, historical untreated records (1992–1993) from the Department of Orthodontics archive were used.

The inclusion criteria for the control group were as follows:

Skeletal Class III malocclusion due to maxillary retrusion (Nperp-A < −1 mm);

Normodivergent vertical pattern (FH/MP within normal limits);

Anterior crossbite with negative overjet;

CVM stages II–III;

Late permanent dentition.

The exclusion criteria were:

Absence of maxillary posterior teeth;

Congenital dental anomalies, impacted or ankylosed teeth;

Periodontal disease;

Previous orthodontic treatment;

Cleft lip and palate.

Control inclusion criteria were comparable to those of the treatment group (Table 1).

The use of a historical control group was considered necessary for ethical reasons, as withholding orthopedic treatment in growing patients with skeletal Class III malocclusion may adversely affect prognosis. To minimize potential bias related to differences in data collection periods, all lateral cephalometric radiographs were obtained using standardized imaging protocols within the same institution, and comparable landmark identification and measurement procedures were applied to both groups. Additionally, magnification differences were standardized prior to analysis to ensure comparability between groups. Furthermore, strict inclusion and exclusion criteria were applied to both groups to enhance baseline comparability and reduce potential differences in population characteristics. However, despite these precautions, the use of a historical control group may still introduce residual bias related to temporal differences in population and clinical conditions.

### 2.3. Appliance Design and Expansion Protocol

**Figure 1:**
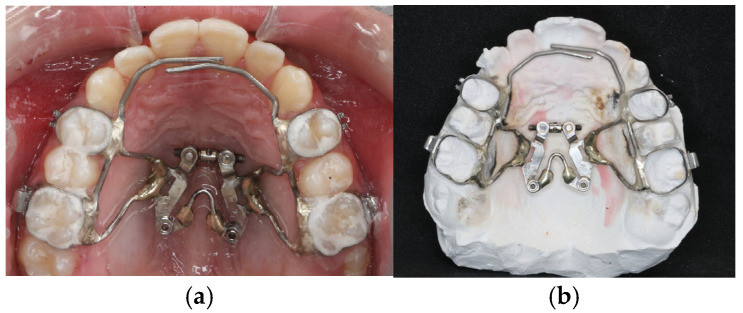
(**a**) The 2-hinged expander prior to cementation. (**b**) The appliance after intraoral cementation.

A tooth-borne 2-hinged expander was banded to the maxillary first premolars and first molars (Figure 1). An expansion screw positioned along the midpalatal suture was used to generate posterior-lateral rotational forces intended to facilitate maxillary mobilization. The double-hinged expander was a custom-made stainless steel appliance fabricated in an orthodontic laboratory. It included an expansion screw (Bestdent, Kaohsiung, Taiwan) positioned along the midpalatal suture, with two posterior hinges near the molar bands and two anterior extension arms (0.045-inch stainless steel wire).

The appliance design incorporated two hinges located in the posterior region, near the molar bands, allowing a posterior displacement of the center of rotation. This configuration enabled a posterior-lateral rotational movement of the maxillary halves during activation, thereby directing forces more effectively toward the circummaxillary sutures rather than concentrating them solely at the midpalatal suture. This biomechanical feature was intended to facilitate maxillary mobilization.

The Alt-RAMEC protocol was initiated one day after cementation. The screw was activated four times daily during the first week (twice in the morning and twice in the evening), producing 1 mm of expansion per day. During the second week, the screw was closed at the same daily rhythm. This expansion–constriction cycle was repeated for 9 consecutive weeks and completed with expansion in the final week (Table 2). After completion of the protocol, the expander was removed and a quad-helix appliance was placed for retention.

### 2.4. Cephalometric Analysis

Standardized lateral cephalograms were obtained at baseline (T0) and immediately after completion of the Alt-RAMEC protocol (T1), corresponding to interval of 9 weeks [34]. All radiographic records were obtained under standardized conditions using the same equipment and patient positioning protocol.

All measurements were performed by a single examiner. To assess intra-examiner reliability, repeated measurements were performed on randomly selected cephalograms, and the results demonstrated high reproducibility.

**Figure 2:**
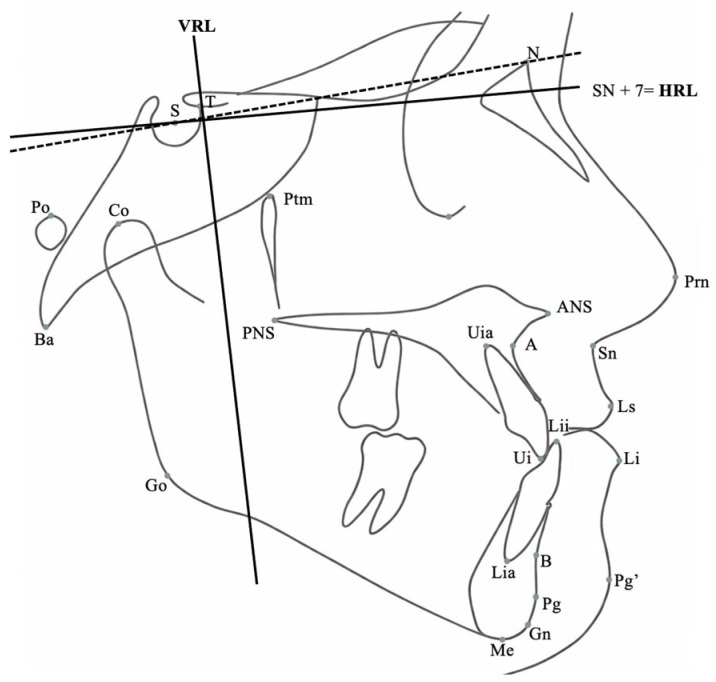
Cephalometric landmarks and horizontal (HRL) and vertical (VRL) reference lines used for skeletal and soft tissue analysis.

**Figure 3:**
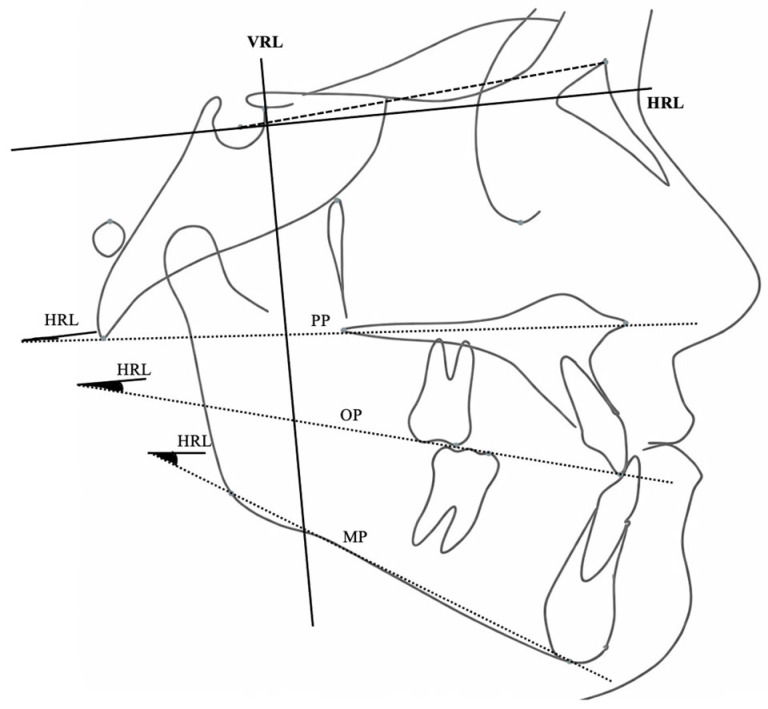
Angular skeletal measurements relative to the horizontal reference line (HRL), including palatal plane (PP), occlusal plane (OP), and mandibular plane (MP).

**Figure 4:**
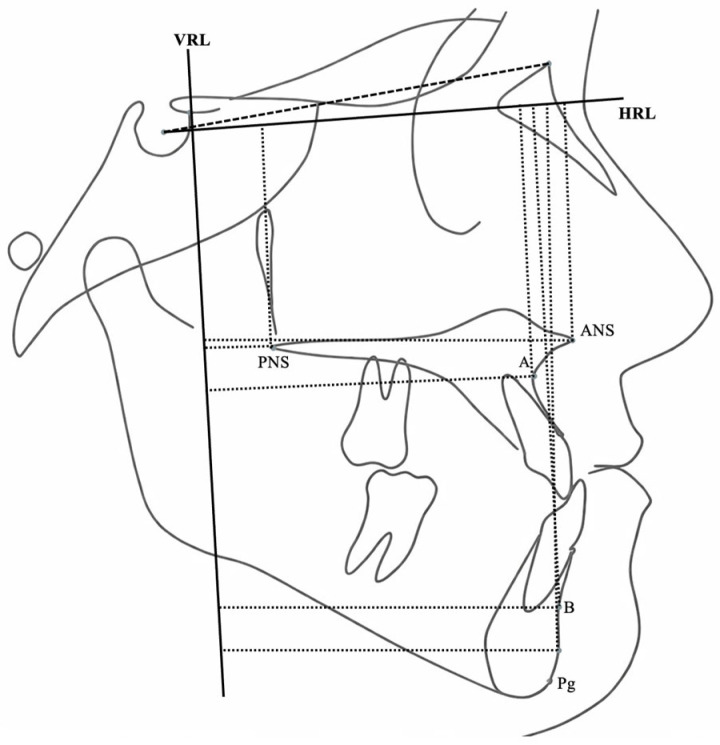
Sagittal and vertical linear skeletal measurements of the maxilla and mandible relative to the Cartesian coordinate system.

**Figure 5:**
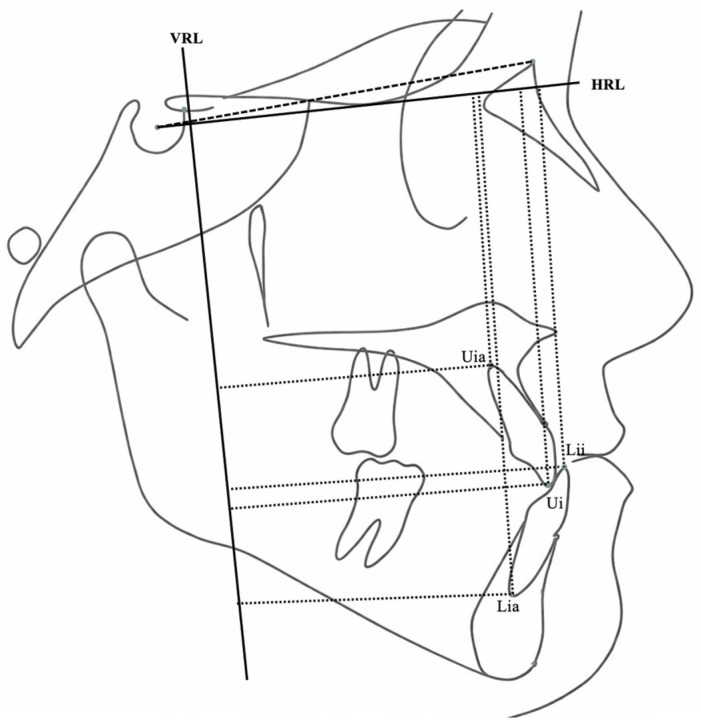
Sagittal and vertical linear dental measurements of the maxilla and mandible relative to the Cartesian coordinate system.

**Figure 6:**
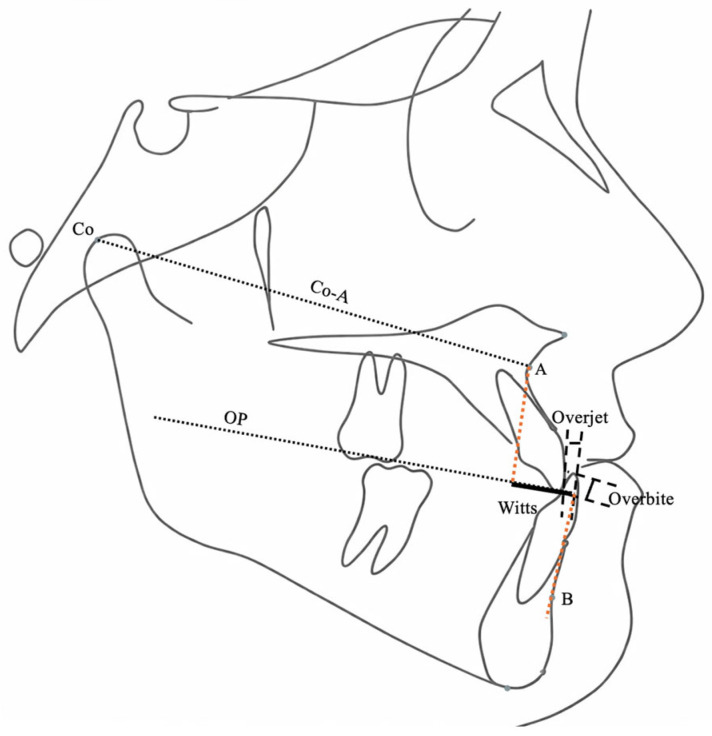
Co–A, Wits appraisal, overjet, and overbite measurements.

**Figure 7:**
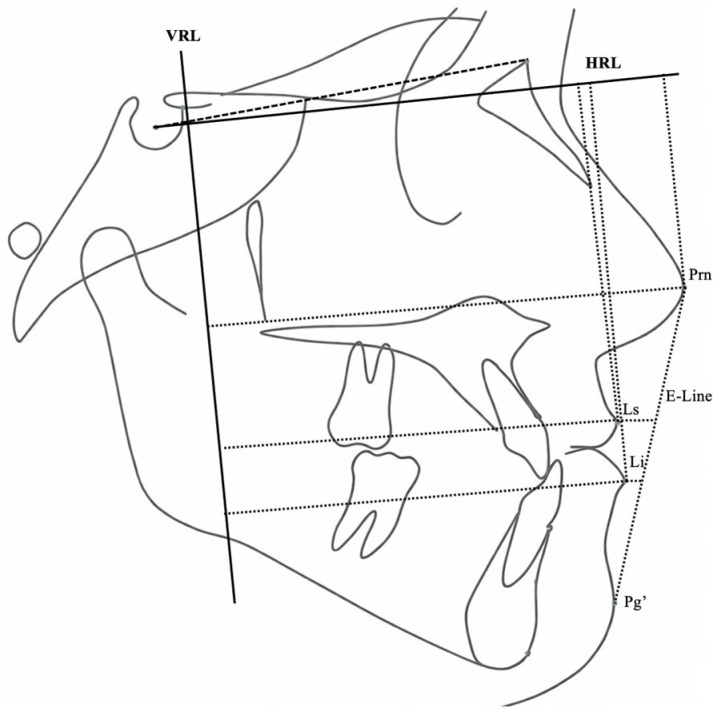
Soft tissue profile linear measurements relative to the HRL, VRL, and E-line.

Sagittal and vertical skeletal variables were evaluated using a Cartesian coordinate system in addition to conventional cephalometric parameters. A horizontal reference line (HRL), constructed by rotating the Sella–Nasion plane 7° downward, served as the *x*-axis. A perpendicular line passing through point T served as the *y*-axis. Linear and angular variables were used to assess skeletal, dental, and soft tissue profile changes (Figure 2, Figure 3, Figure 4, Figure 5, Figure 6 and Figure 7).

The primary outcome variable of this study was defined as the change in anterior maxillary position, assessed by the sagittal displacement of point A relative to the vertical reference line (A–VRL).

Secondary outcome variables included other skeletal, dental, and soft tissue cephalometric measurements, as well as periodontal parameters (PI, GI, PPD, BI, and KGTW).

Magnification differences between groups were standardized before analysis. In addition, to minimize potential methodological differences between historical and contemporary records, all cephalograms were re-analyzed using the same coordinate system, landmark definitions, and measurement protocol by the same examiner.

### 2.5. Periodontal Status Evaluation

The clinical periodontal response of the supporting teeth (maxillary first premolars and first molars) and the remaining maxillary teeth was evaluated at baseline (T0) and immediately after completion of the Alt-RAMEC protocol (T1), corresponding to interval of 9 weeks, by a single calibrated clinician. All clinical assessments were performed under standardized conditions.

Given that periodontal assessments are inherently operator-dependent clinical measurements, the use of a single calibrated clinician helped to ensure consistency and reduce variability; however, intra-examiner reliability for periodontal measurements was not formally assessed.

All measurements were performed using a Williams periodontal probe on six surfaces per tooth (mid-buccal, mesio-buccal, disto-buccal, mid-palatal, mesio-palatal, and disto-palatal).

Plaque Index (PI) and Gingival Index (GI) were recorded according to the criteria described by Silness and Löe [40].

For PI, scores were defined as:

0 = no plaque;
1 = plaque detectable only by probing;
2 = visible plaque accumulation;
3 = abundant plaque or calculus.

For GI, scores were defined as:

0 = healthy gingiva;
1 = mild inflammation without bleeding;
2 = moderate inflammation with bleeding on probing;
3 = severe inflammation with spontaneous bleeding.

Probing pocket depth (PPD) was measured in millimeters. The distance from the gingival margin to the base of the sulcus/pocket was recorded at the same six sites per tooth using gentle probing force (approximately 20–25 g). For each participant, the mean PPD was calculated by averaging all recorded sites.

Bleeding Index (BI) was assessed after gentle probing of the same sites. Each site was observed for approximately 30 s following probing, and the presence or absence of bleeding was recorded. The BI value for each participant was calculated as the percentage of bleeding sites relative to the total number of examined sites.

Keratinized gingival tissue width (KGTW) was measured in millimeters as the distance between the gingival margin and the mucogingival junction at the mid-buccal aspect of each examined tooth. The mean KGTW per participant was calculated for statistical analysis.

For all periodontal parameters, mean values per patient were used for statistical analysis.

### 2.6. Statistical Analysis

Statistical analyses were performed using IBM SPSS Statistics for Windows, Version 20.0 (IBM Corp., Armonk, NY, USA). Normality was assessed with the Shapiro–Wilk test. Because data were not normally distributed, intergroup comparisons were performed using the Mann–Whitney U test, and intragroup changes were evaluated using the Wilcoxon signed-rank test. The level of significance was set at *p* < 0.05, corresponding to a 95% confidence level.

The primary outcome variable of the study was predefined as the change in anterior maxillary position (A–VRL), and the sample size calculation was based on this parameter. Secondary outcomes were considered exploratory; therefore, no formal adjustment for multiple comparisons was applied, and these results should be interpreted with caution.

In addition to T0 and T1 values, change scores (Δ = T1 − T0) were calculated to better represent treatment-related differences. Due to the absence of individual-level paired data in the archived dataset, variability measures for change scores could not be calculated. Furthermore, for several paired and longitudinal analyses, exact *p*-values could not be retrieved from the original statistical outputs; therefore, statistical significance is reported using threshold values (*p* < 0.05, *p* < 0.01, and NS).

Intra-examiner reliability was assessed by re-tracing 22 randomly selected cephalograms. Method error was calculated using Dahlberg’s formula. Intraclass correlation coefficients ranged from 0.94 to 1.00.

## 3. Results

Table 1 presents the baseline age, sex distribution, and cervical vertebral maturation (CVM) stages of the study groups. Intergroup comparisons demonstrated no statistically significant differences in skeletal characteristics at baseline (Table 3).

In addition to T0 and T1 values, treatment-related changes (Δ = T1 − T0) were calculated and considered in the interpretation of the results; however, due to the retrospective nature of the dataset, these values are not presented as a separate column.

### 3.1. Sagittal Maxillary Skeletal Changes

The treatment group demonstrated significant anterior displacement of points A and ANS, whereas the control group showed posterior changes. Both intragroup and intergroup comparisons of T0–T1 changes were statistically significant (*p* < 0.01; Table 4).

Maxillary effective length (Co-A) increased significantly in the treatment group (*p* < 0.05), with no significant change in the control group. Intergroup comparison of Co-A changes was also significant (*p* < 0.05; Table 4).

### 3.2. Vertical Maxillary Skeletal Changes

The PNS-HRL measurement increased significantly in the treatment group compared to the control group (*p* < 0.05; Table 4).

### 3.3. Maxillary Dental Changes

No significant sagittal or vertical positional changes were observed at the incisal edge or apex of the maxillary incisors in either group (Table 4). These findings suggest that the observed treatment effects were predominantly skeletal rather than dentoalveolar in nature.

### 3.4. Mandibular Skeletal and Dental Changes

No significant sagittal or vertical changes were observed in mandibular skeletal measurements or mandibular incisor position in either group (Table 4). This further supports that the treatment effects were primarily related to maxillary skeletal changes, with minimal influence on mandibular structures.

### 3.5. Interdental Changes

Overjet increased significantly, while overbite decreased significantly in the treatment group. No significant changes were observed in the control group. Intergroup differences were significant for both parameters (*p* < 0.01 and *p* < 0.05, respectively; Table 4).

### 3.6. Maxillo-Mandibular Relationship

Wits appraisal increased significantly in the treatment group, with no significant change in the control group. Intergroup comparison of T0–T1 changes was significant (*p* < 0.05; Table 4).

### 3.7. Craniofacial Skeletal Changes

The OP/HRL angle increased significantly in the control group but remained stable in the treatment group. Intergroup differences were significant (*p* < 0.05; Table 4).

### 3.8. Soft Tissue Changes

Prn-VRL and Ls-E values increased significantly in the treatment group (*p* < 0.05), with no significant change in controls. Li-VRL increased significantly in the control group but remained unchanged in the treatment group; however, intergroup differences were not significant.

Vertical soft tissue measurement (Li-HRL) increased significantly in the control group only (*p* < 0.05), with no significant intergroup difference (Table 4).

Overall, the cephalometric findings indicated that the Alt-RAMEC protocol combined with a 2-hinged expander resulted in significant anterior displacement of the maxilla, accompanied by improvements in intermaxillary relationships. These effects were predominantly skeletal in nature, with minimal dentoalveolar and mandibular involvement.

Soft tissue changes were generally modest and consistent with the underlying skeletal alterations, being more evident in the nasal and upper lip regions, while lower lip and vertical parameters showed limited or inconsistent responses.

### 3.9. Periodontal Status Changes

Significant increases were observed in Gingival Index (GI) and probing pocket depth (PPD) for incisors, premolars, and molars after treatment (*p* < 0.05; Table 5). Bleeding Index (BI) also increased significantly in all tooth groups (*p* < 0.01). In contrast, Plaque Index (PI) and keratinized gingival tissue width (KGTW) showed no statistically significant changes (*p* > 0.05).

Although statistically significant increases were observed in gingival index (GI), probing pocket depth (PPD), and bleeding index (BI), the magnitude of these changes remained within clinically acceptable limits. No significant changes were detected in plaque index (PI) or keratinized gingival tissue width (KGTW), suggesting that overall periodontal health was maintained. These findings indicate that the applied protocol did not result in clinically relevant periodontal deterioration despite minor inflammatory changes.

## 4. Discussion

The main finding of this study is that the Alt-RAMEC protocol combined with a 2-hinged expander was associated with anterior maxillary displacement, with effects that appeared to be predominantly skeletal in nature.

These findings suggest that the treatment may primarily induce orthopedic changes, contributing to improvement in intermaxillary relationships without marked dentoalveolar or mandibular alterations. This may support the effectiveness of the protocol in targeting maxillary deficiency in growing patients while potentially minimizing unwanted dental side effects.

By the end of the study, both groups exhibited downward movement of the posterior maxillary region, consistent with normal growth patterns. However, this vertical displacement was greater in the treatment group, which may be attributed to the combined effect of the 2-hinged expander and the Alt-RAMEC protocol.

The absence of significant positional change in the maxillary incisors supports the interpretation that the observed advancement was predominantly skeletal rather than dental. Although overjet increased and a positive sagittal relationship was established, incisor inclination remained stable, further indicating a skeletal effect.

The expansion forces generated at the midpalatal suture are transmitted to the circummaxillary sutures, facilitating mobilization of the maxilla from its cranial attachments. Biederman and Chem proposed that forward displacement of the maxilla through RME depends on the location of the center of rotation, suggesting that forces directed toward the palatomaxillary region favor anterior movement [7]. Decades later, Liou and colleagues demonstrated experimentally and clinically that the 2-hinged expander combined with the Alt-RAMEC protocol effectively redirects forces to this region [7,25].

The primary objective of the 2-hinged expander with Alt-RAMEC is to reduce resistance within the palatomaxillary region by enhancing separation of the palatine bone from the maxilla [7,25,41]. It has been reported that 7–9 weeks of Alt-RAMEC are required to sufficiently open coronally oriented circummaxillary sutures to facilitate maxillary protraction [25,41]. In agreement with these findings, the present study demonstrated effective maxillary mobilization after 9 weeks of Alt-RAMEC.

Melsen and Melsen emphasized that continued transverse palatal growth until approximately 13–15 years of age contributes to favorable orthopedic response [42]. The participants in the present study were at CVM stages II–III, which correspond to the period of active growth and are considered optimal timing for maxillary protraction [25]. This growth phase may explain the magnitude of the skeletal response observed.

The conventional approach suggests that RME followed by facemask (FM) therapy enhances maxillary advancement [6,13,14,15,43]. However, the effectiveness of FM therapy depends largely on patient compliance [25,26]. Alternative approaches have therefore been explored. Huang et al. reported that the 2-hinged expander combined with Alt-RAMEC produced greater anterior maxillary movement compared to Hyrax/Alt-RAMEC, although the Alt-RAMEC protocol itself was considered the principal factor in skeletal advancement [44]. Conversely, Masucci et al. found no significant difference between Alt-RAMEC/FM and RME/FM in short- and medium-term outcomes [33]. In the present study, the untreated control group did not exhibit forward maxillary growth, whereas the treatment group demonstrated significant anterior displacement, supporting the skeletal effectiveness of the 2-hinged expander combined with Alt-RAMEC.

These similarities and discrepancies with previous studies may be attributed to differences in appliance design, force application mechanics, duration of the Alt-RAMEC protocol, and patient growth status. In particular, the incorporation of hinges in the expander design may alter force vectors and enhance anterior maxillary displacement compared to conventional expanders. Additionally, variations in study design, including sample characteristics and observation periods, may further contribute to the heterogeneity of reported outcomes.

Maxillary advancement was accompanied by an increase in overjet and improvement in Wits appraisal, as well as forward positioning of the upper lip, consistent with previous reports [26,29,30,31,45]. These findings indicate that skeletal changes were reflected in soft tissue profile improvement.

Periodontal response should be interpreted cautiously, as individual variability plays a significant role in periodontal health [46]. Previous CBCT studies have reported thinning of buccal cortical bone following RME and alterations in alveolar bone thickness after Alt-RAMEC [35]. Both tooth-borne and tissue-borne expanders may increase plaque retention and predispose to gingival inflammation. In the present study, although plaque levels remained stable, GI, BI, and PPD increased significantly immediately after treatment. However, the magnitude of these changes was limited. Given the absence of a control group for periodontal parameters and the short-term follow-up, it is not possible to determine whether these changes represent transient inflammatory responses or more persistent alterations. Therefore, these findings should be interpreted with caution. To date, limited data exist regarding the soft tissue periodontal effects of the 2-hinged expander combined with Alt-RAMEC. Therefore, this study provides preliminary evidence regarding the immediate periodontal response to this protocol.

This study has several limitations. First, the prospective treatment group was compared with a historical untreated control group. Although strict inclusion criteria were applied to ensure baseline comparability, the absence of a contemporaneous prospective control group may introduce potential bias related to temporal differences in growth patterns, radiographic acquisition conditions, and population characteristics.

In addition, periodontal parameters were evaluated only in the treatment group, and no untreated control data were available for comparison. Therefore, it is not possible to fully distinguish treatment-related changes from potential time-related or oral hygiene-related variations. Furthermore, intra-examiner reliability for periodontal measurements was not formally assessed. Although all measurements were performed by a single calibrated clinician under standardized conditions, the operator-dependent nature of clinical periodontal assessments may introduce measurement variability.

Second, although the initial sample size calculation indicated a minimum of 20 participants, only 15 patients in the treatment group completed the study due to loss to follow-up. This reduction may have decreased the statistical power of the study and may limit the generalizability of the findings. In particular, the reduced sample size may have affected the ability to detect statistically significant differences for some variables, thereby increasing the risk of type II error. Therefore, non-significant findings should be interpreted with caution, as they may not necessarily indicate the absence of a true effect.

Third, the follow-up period was limited to the immediate post-treatment phase. Therefore, the long-term stability of the observed skeletal, dental, and periodontal changes could not be evaluated.

Fourth, exact *p*-values could not be consistently retrieved from the archived statistical outputs, and statistical significance was therefore reported using threshold notation. While this approach is commonly used, it may limit the precision of statistical interpretation.

Finally, three-dimensional imaging could not be performed due to ethical restrictions regarding CBCT use in growing individuals. Although standardized lateral cephalograms were obtained, two-dimensional imaging may limit the precision of skeletal assessment compared with CBCT-based evaluation. In particular, lateral cephalometry does not allow comprehensive evaluation of transverse and rotational components of maxillary displacement. Therefore, the skeletal findings of the present study should be interpreted within the limitations of two-dimensional analysis.

Despite these limitations, the findings of this study have clinical relevance, suggesting that the Alt-RAMEC protocol combined with a 2-hinged expander may be a useful orthopedic approach for the management of maxillary deficiency in growing patients, potentially providing skeletal correction with limited dentoalveolar side effects and without clinically relevant periodontal deterioration.

Future studies with larger sample sizes, prospective controlled designs, longer follow-up periods, and three-dimensional imaging modalities are needed to confirm the present findings and to better evaluate the long-term stability and clinical effects of this treatment protocol.

## 5. Conclusions

The 2-hinged expander combined with a 9-week Alt-RAMEC protocol was associated with anterior maxillary displacement in adolescents with skeletal Class III malocclusion characterized by maxillary retrusion.

Maxillary advancement appeared to occur predominantly through skeletal effects, with no significant proclination of the maxillary incisors, suggesting limited dental compensation.

Mandibular and craniofacial skeletal changes were generally consistent with growth-related patterns observed in the untreated control group.

Short-term increases in gingival index, bleeding index, and probing depth were observed immediately after treatment, indicating a temporary worsening of selected periodontal soft-tissue parameters. However, due to the absence of longitudinal follow-up data, it remains unclear whether these changes are reversible or clinically significant. Therefore, further long-term studies are required to clarify the periodontal effects of this treatment protocol.

## Figures and Tables

**Table 1:** Baseline age and CVM stage distribution of the study groups.

	Treatment Group (n = 15)			Control Group (n = 27)		
	Age (Years), Mean ± SD	CVM II	CVM III	Age (Years), Mean ± SD	CVM II	CVM III
Girls (n = 6/7)	12.6 ± 1.42	3	3	12.5 ± 1.54	0	7
Boys (n = 9/20)	13.1 ± 1.92	5	4	12.6 ± 1.45	13	7

Values for girls and boys are presented separately for the treatment (n = 15) and control (n = 27) groups. CVM: Cervical vertebral maturation stage. n: number of participants. SD: standard deviation.

**Table 2:** Alt-RAMEC activation protocol over the 9-week treatment period.

Week	Protocol Phase	Activation Schedule	Daily Activation
1	Expansion	2 turns morning + 2 turns evening	1 mm/day
2	Constriction	2 turns morning + 2 turns evening	1 mm/day
3	Expansion	2 turns morning + 2 turns evening	1 mm/day
4	Constriction	2 turns morning + 2 turns evening	1 mm/day
5	Expansion	2 turns morning + 2 turns evening	1 mm/day
6	Constriction	2 turns morning + 2 turns evening	1 mm/day
7	Expansion	2 turns morning + 2 turns evening	1 mm/day
8	Constriction	2 turns morning + 2 turns evening	1 mm/day
9	Expansion	2 turns morning + 2 turns evening	1 mm/day

**Table 3:** Baseline skeletal characteristics of the treatment and control groups (T0).

Measurements	Treatment Group (T0) Mean ± SD	Control Group (T0) Mean ± SD	*p*
Nperp–A, mm	−1.00 ± 1.91	−2.14 ± 4.10	NS
Co–A, mm	85.67 ± 7.29	88.71 ± 6.60	NS
B–VR, mm	63.33 ± 7.76	61.14 ± 11.81	NS
Pg–Nperp, mm	5.59 ± 6.63	4.43 ± 3.82	NS
Wits appraisal, mm	−0.67 ± 3.09	0.57 ± 1.40	NS
FH/MP, °	22.46 ± 3.38	25.14 ± 5.58	NS
NBa/Ptm–Gn, °	90.9 ± 3.20	91.57 ± 4.08	NS

NS: Not significant. Exact *p*-values were not consistently available from the original statistical outputs; therefore, statistical significance is reported using threshold notation.

**Table 4:** Intragroup and intergroup comparisons of cephalometric changes between T0 and T1.

Measurement	Treatment Group (T0) (Mean ± SD)	Treatment Group (T1) (Mean ± SD)	Control Group (T0) (Mean ± SD)	Control Group (T1) (Mean ± SD)	*p*
Sagittal Maxillary Skeletal					
A–VRL (mm)	59.37 ± 4.01	61.93 ± 3.98	62.71 ± 6.32	61.14 ± 8.12	**
ANS–VRL (mm)	63.80 ± 3.70	66.20 ± 8.29	69.00 ± 5.86	67.57 ± 8.54	**
PNS–VRL (mm)	17.13 ± 4.10	17.40 ± 3.40	16.36 ± 4.84	17.00 ± 5.16	NS
Co–A (mm)	85.67 ± 7.29	88.70 ± 5.37	88.71 ± 6.60	90.21 ± 4.98	*
Vertical Maxillary Skeletal					
A–HRL (mm)	45.57 ± 5.60	46.70 ± 5.93	49.86 ± 3.18	50.79 ± 4.18	NS
ANS–HRL (mm)	40.97 ± 5.45	42.97 ± 5.38	43.71 ± 4.19	47.71 ± 3.82	NS
PNS–HRL (mm)	40.23 ± 4.07	41.80 ± 4.32	40.93 ± 7.66	41.93 ± 7.37	*
Sagittal Maxillary Dental					
Ui–VRL (mm)	63.00 ± 5.89	64.30 ± 4.69	65.79 ± 6.87	66.71 ± 10.81	NS
Uia–VRL (mm)	54.70 ± 4.12	54.90 ± 3.27	59.00 ± 10.83	58.93 ± 9.10	NS
Vertical Maxillary Dental					
Ui–HRL (mm)	67.60 ± 7.61	68.70 ± 6.82	71.43 ± 6.65	73.79 ± 6.04	NS
Uia–HRL (mm)	51.20 ± 13.62	52.70 ± 12.84	50.57 ± 8.54	52.36 ± 7.87	NS
Sagittal Mandibular Skeletal					
B–VRL (mm)	63.33 ± 7.76	61.37 ± 5.77	61.14 ± 11.81	59.36 ± 11.10	NS
Pg–VRL (mm)	63.03 ± 7.32	61.43 ± 7.48	61.07 ± 12.10	61.43 ± 11.98	NS
Vertical Mandibular Skeletal					
B–HRL (mm)	83.63 ± 9.32	86.27 ± 7.60	86.07 ± 11.03	87.86 ± 6.39	NS
Pg–HRL (mm)	98.33 ± 10.85	100.60 ± 9.27	99.00 ± 17.11	102.30 ± 7.81	NS
Sagittal Mandibular Dental					
Li–VRL (mm)	64.93 ± 4.76	65.40 ± 5.11	63.93 ± 10.58	65.36 ± 11.21	NS
Lia–VRL (mm)	55.73 ± 7.59	56.73 ± 7.03	46.00 ± 17.35	53.00 ± 13.89	NS
Vertical Mandibular Dental					
Li–HRL (mm)	66.33 ± 6.97	68.20 ± 6.30	71.57 ± 7.11	73.00 ± 6.24	NS
Lia–HRL (mm)	86.73 ± 7.38	88.67 ± 6.76	92.00 ± 8.08	93.57 ± 8.16	NS
Interdental					
Overjet (mm)	−2.57 ± 0.86	−0.43 ± 1.51	0.14 ± 0.38	0.29 ± 0.76	**
Overbite (mm)	2.93 ± 1.44	1.63 ± 1.06	0.14 ± 0.38	1.36 ± 1.18	*
Maxillo-Mandibular					
Wits (mm)	−3.13 ± 2.88	−0.67 ± 3.09	0.29 ± 0.76	0.57 ± 1.40	*
Skeletal Craniofacial					
PP/HRL (°)	3.80 ± 2.46	3.87 ± 1.96	8.14 ± 6.20	7.71 ± 3.59	NS
OP/HRL (°)	9.53 ± 3.38	10.60 ± 4.70	22.71 ± 9.45	24.14 ± 7.36	NS
MP/HRL (°)	23.87 ± 6.20	25.20 ± 5.53	22.71 ± 9.45	24.14 ± 7.36	NS
Sagittal Soft Tissue Profile					
Prn–VRL (mm)	89.07 ± 5.51	90.80 ± 5.97	95.00 ± 3.32	96.57 ± 6.24	*
Ls–E line (mm)	−6.93 ± 2.89	−5.60 ± 2.75	−3.50 ± 3.14	−3.93 ± 5.47	*
Ls–VRL (mm)	76.87 ± 5.13	78.50 ± 5.10	78.79 ± 6.95	76.64 ± 10.17	*
Li–VRL (mm)	77.40 ± 5.90	77.00 ± 8.49	75.71 ± 12.81	80.00 ± 9.95	NS
Vertical Soft Tissue Profile					
Prn–HRL (mm)	35.73 ± 7.46	35.47 ± 5.79	45.86 ± 4.46	46.57 ± 4.46	NS
Ls–HRL (mm)	57.73 ± 6.70	59.07 ± 6.27	64.14 ± 9.53	63.29 ± 5.91	NS
Li–E (mm)	−0.33 ± 3.81	−1.13 ± 3.18	−0.57 ± 4.65	0.71 ± 3.90	NS
Li–HRL (mm)	73.73 ± 7.46	75.47 ± 7.04	78.29 ± 9.89	84.00 ± 7.57	NS

Intragroup comparisons were performed using the Wilcoxon signed-rank test. Intergroup comparisons were analyzed using the Mann–Whitney U test. In addition to T0 and T1 values, change scores (Δ = T1 − T0) were calculated and considered in the interpretation of the results; however, due to the retrospective nature of the dataset, these values are not presented as a separate column. Significance level: * *p* < 0.05, ** *p* < 0.01, NS: Not significant. Exact *p*-values were not consistently available from the original statistical outputs.

**Table 5:** Comparison of Soft Tissue Periodontal Parameters of Maxillary Incisors, First Premolars, and First Molars at T0 and T1.

Parameter	Incisors T0 (Mean ± SD)	Incisors T1 (Mean ± SD)	*p*	Premolars T0 (Mean ± SD)	Premolars T1 (Mean ± SD)	*p*	Molars T0 (Mean ± SD)	Molars T1 (Mean ± SD)	*p*
PI	0.61 ± 0.47	0.73 ± 0.32	NS	0.71 ± 0.47	0.83 ± 0.32	NS	0.81 ± 0.47	0.83 ± 0.32	NS
GI	0.54 ± 0.12	0.78 ± 0.31	*	0.63 ± 0.12	0.77 ± 0.31	*	0.78 ± 0.12	0.87 ± 0.31	*
PPD (mm)	1.18 ± 0.22	1.33 ± 0.28	*	1.17 ± 0.22	1.55 ± 0.28	*	1.23 ± 0.22	1.37 ± 0.28	*
BI (%)	25.5 ± 15.03	39.87 ± 19.08	**	28.9 ± 16.1	44.2 ± 20.3	**	27.4 ± 14.8	42.6 ± 18.7	**
KGTW (mm)	7.80 ± 1.52	8.13 ± 1.19	NS	6.07 ± 1.67	7.13 ± 1.46	NS	6.13 ± 1.42	7.13 ± 1.25	NS

Abbreviations: PI, Plaque Index; GI, Gingival Index; PPD, Probing Pocket Depth; BI, Bleeding Index; KGTW, Keratinized Gingival Tissue Width; SD, Standard Deviation; NS, Not Significant. In addition to T0 and T1 values, change scores (Δ = T1 − T0) were calculated and considered in the interpretation of the results; however, due to the retrospective nature of the dataset, these values are not presented as a separate column. Significance level: * *p* < 0.05; ** *p* < 0.01. Exact *p*-values were not consistently available from the original statistical outputs.

## Data Availability

The data presented in this study are not publicly available due to ethical and privacy restrictions, as they are derived from anonymized patient radiographic records obtained from institutional archives. Data may be made available from the corresponding author upon reasonable request and with permission of the relevant ethics committee.

## Abbreviations

RME	Rapid Maxillary Expansion (RME)
Alt-RAMEC	Alternate Rapid Maxillary Expansion and Construction
FM	Face Mask
CT	Computerized Tomography
CBCT	Cone Beam Computerized Tomography
HRL	Horizontal Reference Line
VRL	Perpendicular Line
CVM	Cervical Vertebrae Maturation
PI	Plaque Index
GI	Gingival Index
PPD	Periodontal Pocket Depth
BI	Bleeding Index
KGT	Keratinized Gingival Tissue
